# Cross-talk and transitions between multiple environments in an attractor neural network model of the hippocampus

**DOI:** 10.1186/1471-2202-14-S1-O15

**Published:** 2013-07-08

**Authors:** Sophie Rosay, Rémi Monasson

**Affiliations:** 1 Laboratory of Theoretical Physics, CNRS & Ecole Normale Supérieure, Paris, 75005, France

## 

Place cells are neurons in the hippocampus whose activity depends on the animal's location in space and are therefore thought to be crucial for spatial representation [1]. Based on the assumption that CA3 works as an attractor neural network [2] models have shown that spatially-localized attractors, corresponding to different 'environments' or 'spatial maps', can be encoded in one network [2,3]. Transitions and cross-talks between attractors coding for different maps remain, however, poorly understood.

**Figure 1:**
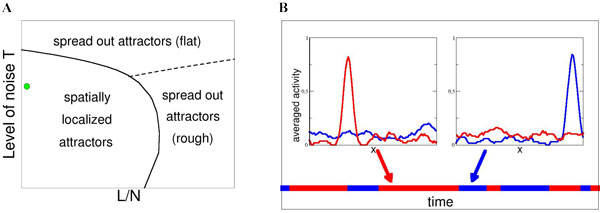
**Properties of the model (1D-space with w = 0**.05, 10% of active neurons). **A**. Sketch of the phase diagram. For moderate T and L the network activity is spatially localized in one of the stored maps and encode a specific location in space. For high T (strong noise) or high L (strong map interference) the activity profile spreads over the whole space, either uniformly or with rough, interference-induced ripples. **B**. Transitions between two stored maps (symbolized with blue and red colors) during a Monte Carlo simulation with N = 1000 neurons ; values of T and L correspond to the green spot in panel A. Insets : place-cell activity vs. place-field center locations in blue and red maps, for two instants indicated by the arrows.

Motivated by a recent experiment showing bistability between competing spatial representations, paced by theta waves [4] we propose a recurrent model network, whose synaptic connections J_ij _sum up contributions coming from all the environments according to : 1. The contribution to J_ij _due to an environment vanish when the centers of the place fields of cells i & j are further away than some cut-off distance w; 2. Place fields are randomly remapped from one environment to the other. Using tools and concepts from the statistical physics of disordered systems we have solved the model and show that the network can be in one of three regimes, depending on the level of noise in the neural dynamics, T, and the number of environments, L (Figure 1A). In particular, we have found the maximal values of T and L (given the other parameters of the model e.g. the number of place cells, the average firing rate, ...) such that,spatially-localized and environment-specific activity is possible. In addition we have observed the presence of spontaneous, i.e. in the absence of external input, dynamical transitions from the activity localized in one map to the activity representative of another environment (Figure 1B). Those transitions are strongly reminiscent of those experimentally observed in [4]. The statistical features of the transitions and their dependence on the parameters of the model can be understood in great analytical details.
